# Predicting Adaptations to Resistance Training Plus Overfeeding Using Bayesian Regression: A Preliminary Investigation

**DOI:** 10.3390/jfmk6020036

**Published:** 2021-04-21

**Authors:** Robert W. Smith, Patrick S. Harty, Matthew T. Stratton, Zad Rafi, Christian Rodriguez, Jacob R. Dellinger, Marqui L. Benavides, Baylor A. Johnson, Sarah J. White, Abegale D. Williams, Grant M. Tinsley

**Affiliations:** 1 Energy Balance & Body Composition Laboratory, Department of Kinesiology & Sport Management, Texas Tech University, Lubbock, TX 79409, USA; robert.smith@ttu.edu (R.W.S.); patrick.harty@ttu.edu (P.S.H.); matthew.stratton@ttu.edu (M.T.S.); christian.rodriguez@ttu.edu (C.R.); jacob.dellinger@ttu.edu (J.R.D.); marqui.benavides@ttu.edu (M.L.B.); baylor.johnson@ttu.edu (B.A.J.); sarah.white@ttu.edu (S.J.W.); abegale.williams@ttu.edu (A.D.W.); 2 NYU Langone Medical Center, New York, NY 10016, USA; zad@lesslikely.com

**Keywords:** energy surplus, hypertrophy, weight gain, bulking, calorie surplus, muscle gain

## Abstract

Relatively few investigations have reported purposeful overfeeding in resistance-trained adults. This preliminary study examined potential predictors of resistance training (RT) adaptations during a period of purposeful overfeeding and RT. Resistance-trained males (*n* = 28; *n* = 21 completers) were assigned to 6 weeks of supervised RT and daily consumption of a high-calorie protein/carbohydrate supplement with a target body mass (BM) gain of ≥0.45 kg·wk^−1^. At baseline and post-intervention, body composition was evaluated via 4-component (4C) model and ultrasonography. Additional assessments of resting metabolism and muscular performance were performed. Accelerometry and automated dietary interviews estimated physical activity levels and nutrient intake before and during the intervention. Bayesian regression methods were employed to examine potential predictors of changes in body composition, muscular performance, and metabolism. A simplified regression model with only rate of BM gain as a predictor was also developed. Increases in 4C whole-body fat-free mass (FFM; (mean ± SD) 4.8 ± 2.6%), muscle thickness (4.5 ± 5.9% for elbow flexors; 7.4 ± 8.4% for knee extensors), and muscular performance were observed in nearly all individuals. However, changes in outcome variables could generally not be predicted with precision. Bayes R^2^ values for the models ranged from 0.18 to 0.40, and other metrics also indicated relatively poor predictive performance. On average, a BM gain of ~0.55%/week corresponded with a body composition score ((∆FFM/∆BM)*100) of 100, indicative of all BM gained as FFM. However, meaningful variability around this estimate was observed. This study offers insight regarding the complex interactions between the RT stimulus, overfeeding, and putative predictors of RT adaptations.

## 1. Introduction

Increasing body mass (BM), particularly due to fat-free mass (FFM) accretion, is a common training goal for athletes. This process, colloquially referred to as “bulking,” is frequently initiated in the off-season for competitive athletes and consists of a designated period of intentional overfeeding in conjunction with high-volume resistance training (RT). Although the strategic utilization of a hypercaloric diet has long been employed by practitioners, early research support for this practice can be traced to work detailing the anabolic effects of a positive energy balance [1]. Today, athletes seeking to increase BM and FFM modulate their training practices and employ various nutritional and dietary supplement strategies to promote tissue growth and associated improvements in muscular performance. A commonly held belief amongst practitioners is that a slower rate of BM gain, elicited by a modest caloric surplus, allows for an increased proportion of BM to be gained as FFM. Possible support for this idea comes from a limited amount of research reporting that an energy surplus of ≤800 kcal/d during RT in resistance-trained individuals resulted in ~100% of the increased BM to be attributable to FFM over durations of approximately eight weeks [2,3]. The current body of evidence indicates a likely influence of participant training status on RT adaptations in response to a given magnitude of energy surplus. For example, Rozenek et al. [4] demonstrated that large caloric surpluses of ~2000 kcal/d from protein/carbohydrate or carbohydrate supplements led to increases in FFM, but not FM, in relatively untrained individuals performing RT. In contrast, another investigation found that overfeeding with >1000 kcal/d of protein/carbohydrate did not improve FFM accretion during RT in resistance-trained individuals [5]. While these investigations provide preliminary information regarding overfeeding in conjunction with RT, the relative dearth of research and limitations in the methods used to evaluate body composition have precluded definitive conclusions regarding rate of BM gain prescriptions to promote superior training adaptations. The few previous investigations examining the impact of overfeeding with RT have estimated the changes in body composition using dual-energy X-ray absorptiometry (DXA) [5,6], air displacement plethysmography (ADP) [7,8,9], hydrostatic weighing [4], and ultrasonography [2]. Although these methods are typically viewed as acceptable laboratory methods, they have notable limitations as compared to a criterion multi-component model, such as the 4-component (4C) model [10,11,12]. These limitations, such as assumptions about the density and hydration of FFM, have been documented to introduce potentially concerning levels of error in both the general population [13,14] and resistance-trained individuals [15,16]. As such, there is a need for a critical evaluation of body composition changes during overfeeding using a criterion 4C model, which takes into account the mass, volume, water, and bone mineral content of the body.

Though determining the optimal rate of BM gain to promote preferential FFM accretion and other RT adaptations has important implications for researchers and practitioners, there are challenges to quantifying it using standard experimental designs. This research question can be addressed by prescribing and monitoring different rates of BM gain in distinct groups; however, a number of real-world limitations limit the usefulness of this approach. Individual variation in a variety of factors such as the ease of maintaining an energy surplus, metabolic rate adaptations, spontaneous physical activity, the magnitude of energy surplus needed for a particular amount of BM gain, genetics, and the overall propensity to gain BM likely make adherence to a precise rate of BM gain very difficult for participants. These factors and any resultant “noncompliance” with the prescribed rate of BM gain would likely be magnified when attempting to compare small differences in caloric surpluses and rates of BM gain (e.g., 0.25 vs. 0.5 kg per week) across an extended intervention. Due to the aforementioned individual variability in factors pertinent to BM accretion, it would be expected that a notable range of BM gain would be observed even when individuals are provided with a standardized intervention to elicit an increase in BM. As such, the natural variability in responses to a lifestyle intervention designed to increase BM would allow for a practical evaluation of whether the rate of BM gain is related to the composition of the gained BM. This approach offers several advantages, including the removal of dichotomy in describing rates of BM gain (i.e., “slow” versus “fast”) in favor of an evaluation of the rate of BM gain as a continuous variable and an improved likelihood of participants adhering to the study intervention.

Importantly, many other putative predictor variables beyond the rate of BM gain may help explain RT adaptations. Examples include training status, intake of specific nutrients, current and former RT program variables, current body composition, and spontaneous adaptations in physical activity and metabolism during overfeeding. While each of these could potentially influence RT adaptations, incomplete information is available concerning their relative importance. Thus, the purpose of this preliminary study was to examine the relationship between potential predictors of RT adaptations and changes in body composition, muscular performance, and metabolism during a 6-week period of purposeful overfeeding and RT. A special emphasis was placed on the potential importance of the rate of BM gain. While it was hypothesized that a slower rate of BM gain may elicit more favorable body composition changes, additional hypotheses concerning the relative contributions of different predictor variables for various RT adaptations were not made due to the exploratory nature of this investigation.

## 2. Materials and Methods

### 2.1. Participants and Study Design

Individuals were eligible to participate in this intervention if they were between the ages of 18 and 40, male, generally healthy, weight-stable (defined as no change in BM > 2.3 kg in the past 3 months), resistance-trained (defined as performing resistance exercise 2–5 d·wk^−1^ for ≥6 months prior to screening), able to bench press ≥1.0 × BM and leg press ≥ 2.0 × BM during baseline one-repetition maximum (1RM) assessments, and willing to abstain from consumption of any supplement beyond a standard multivitamin or those provided as part of the study. Participants who had previously administered anabolic-androgenic steroids, based on self-report, or who had consumed creatine-containing supplements within the past month, with a total dosage > 10 g/week, were ineligible. All subjects gave their informed consent for inclusion before they participated in the study. The study was conducted in accordance with the Declaration of Helsinki, and the protocol was approved by the Texas Tech University Institutional Review Board (Project identification code: IRB2019-356). This data collection was prospectively registered on clinicaltrials.gov (ClinicalTrials.gov Identifier: NCT04069351; https://clinicaltrials.gov/ct2/show/NCT04069351; first posted: 28 August 2019; first participant enrolled: 18 September 2019).

**Figure 1:**
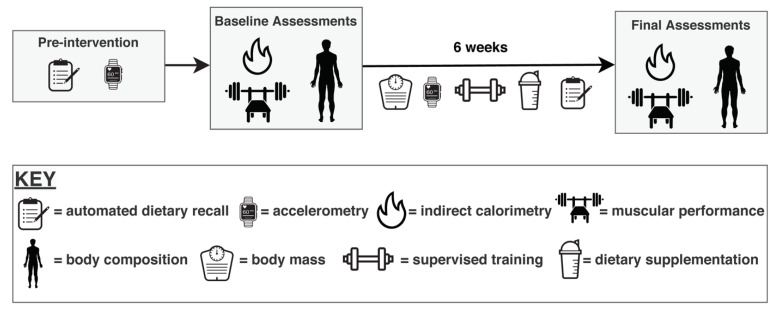
Study Design. Participants performed six weeks of supervised resistance training in conjunction with purposeful overfeeding. Dietary and physical activity assessments were performed prior to the intervention as well as during the intervention. Before and after the intervention, assessments of body composition via four-component model, metabolism via indirect calorimetry, and muscular performance via one-repetition maximum and repetitions to failure on the bench press and leg press exercises were performed.

A total of 32 individuals consented to participate in the study. Four individuals did not meet muscular performance screening criteria and were therefore unable to begin the intervention. The remaining 28 were included in the imputed data sets for this analysis, although five participants dropped out of the study voluntarily for reasons unrelated to the study, and two participants were withdrawn during the intervention for lack of compliance with the supervised RT program. Therefore, twenty-one participants completed the entire study. Participants were assigned to complete 6 weeks of supervised RT, performed 3 d·wk^−1^, and instructed to consume a high-calorie protein/carbohydrate supplement daily. All participants were encouraged to gain ≥0.45 kg·wk^−1^ and were weighed before RT sessions to promote compliance. Before and after the intervention, laboratory assessments were performed to evaluate body composition, metabolism, and exercise performance (Figure 1).

### 2.2. Intervention

#### 2.2.1. Dietary Program

Participants were instructed to maintain their habitual dietary intake while consuming an additional high-calorie protein/carbohydrate supplement provided by the research personnel. Participants were provided with a half-serving (5.5 g fat, 123.5 g carbohydrate, 26 g protein, ~647.5 kcal) of Super Mass Gainer^TM^ (Dymatize Enterprises, LLC., Dallas, TX, USA) to promote BM gain. On training days, the supplement was consumed in the laboratory immediately following the RT session and under researcher supervision. On non-training days, participants were allowed to consume the supplement at their preferred time. All participants were assigned to gain a minimum of 0.45 kg (1 pound) per week, with the total desired BM gained during the 6-week intervention being a minimum of 2.7 kg (6 pounds). BM was assessed in the laboratory prior to RT sessions in order to crudely monitor compliance with targeted BM gain based on the weekly average BM. If participants were unable to meet the prescribed BM gain goal, research personnel provided advice to promote increased energy intake through greater food consumption or increased dosage of the dietary supplement to one full serving each day.

#### 2.2.2. Resistance Training Program

Each participant completed six weeks of supervised RT in conjunction with the assigned supplementation program. The RT program was a progressive regimen designed to induce muscular hypertrophy. Three RT sessions took place within the research laboratory each week, and all major muscle groups were trained two times per week. Trainers who were Certified Strength and Conditioning Specialists (CSCS) or certified personal trainers supervised all training sessions and provided strong verbal encouragement and feedback to participants throughout each training session. Each week of training was broken down into a lower-body training session on Day 1, an upper-body session on Day 2, and a full-body session on Day 3 (Table 1). The majority of exercises were completed with the use of free-weights (barbells and dumbbells) or select weight machines (e.g., hip sled, leg extension, leg curl). Larger muscle groups, primarily targeted through multi-joint barbell movements, were prioritized in the program due to the goal of maximal FFM accretion. Exercise intensity was varied through the training based on repetitions in reserve (RIR) [17]. Weeks 1 and 4 were set at a training intensity of 2 RIR, Weeks 2 and 5 were set at an intensity of 1 RIR, and Weeks 3 and 6 were set at an intensity of 0 RIR. An RIR of 0 meant that each set was taken to momentary muscular failure, and the load was adjusted as necessary to ensure momentary muscular failure within the specified repetition range. Workout logs were completed throughout the study in order to assess relevant training metrics.

### 2.3. Muscular Performance Assessments

Muscular performance was assessed at baseline and post-intervention. Prior to assessments of muscular performance, participants were instructed to follow their preferred food and fluid intake patterns. The assessment began with a 5-min general warm up period prior to the bench press exercise. Upon completion of the 5-min warm up, participants completed the muscular strength and endurance assessment on the bench press exercise. The same warm up procedure was followed for the hip sled exercise. For both exercises, performance was evaluated by the 1RM protocol, followed by repetitions to failure with 70% of the baseline 1RM. The 1RM testing protocol was based on the recommendations of the National Strength and Conditioning Association [18]. Participants first performed a set of 8–10 repetitions with a load <40–60% of their estimated 1RM, then a second set of 8–10 repetitions with a load corresponding to 40–60% of their estimated 1RM, followed by 3–5 repetitions at 60–80% of their estimated 1RM. Participants then completed 2–3 repetitions at ~80–90% of their estimated 1RM. The 1RM attempts were then performed, with the goal of obtaining the 1RM within three to five attempts. The maximal weight lifted with proper form was recorded as the participant’s 1RM. Rest times ranged from one to three minutes between the pre-1RM sets, and a 3-min rest period was implemented between each 1RM attempt. A recent systematic review indicated high reliability of 1RM assessments, with a median ICC of 0.97 and a median coefficient of variation (CV) of 4.2% [19]. After the 1RM was obtained on a given exercise, participants rested for three minutes before completing a repetitions-to-failure (RTF) test. For the RTF test, each participant was instructed to perform as many repetitions as possible with a load corresponding to 70% of the baseline 1RM. Participants were instructed to perform repetitions quickly while maintaining proper technique and full range of motion. Research personnel counted the number of successful repetitions with a clicker and the repetitions were written down immediately after the test ended. Upon volitional muscle failure or failure to execute a repetition with proper technique, the RTF test was considered complete. For all tests, participants received strong verbal encouragement from study personnel.

### 2.4. Laboratory Assessments

#### 2.4.1. Initial Procedures

At baseline and post-intervention, participants reported to the laboratory after abstention from eating, drinking, exercising and utilizing caffeine or nicotine for ≥8 h. Each participant was interviewed to confirm adherence to these pre-assessment restrictions. Participants wore light athletic clothing and removed all metal and accessories from the body prior to testing. Each participant voided his bladder to provide a urine sample for assessment of urine specific gravity (USG) with a digital refractometer (PA201X-093, Misco, Solon, OH, USA). After voiding, each participant’s height was determined via mechanical stadiometer (Seca 769, Hamburg, Germany).

#### 2.4.2. 4-Component Model Body Composition Analysis

Body composition was assessed using a 4C model at baseline and post-intervention. This model utilized assessments via ADP, DXA, and bioimpedance spectroscopy (BIS). All equipment was calibrated according to manufacturer recommendations each day prior to use. Participants wore spandex compression shorts and a swim cap for ADP assessments and light athletic clothing with no metal or accessories for DXA and BIS. ADP (Bod Pod, Cosmed USA, Concord, CA, USA) was performed to estimate body volume (BV) according to manufacturer recommendations utilizing estimated thoracic volume. The BM estimate used in the 4C model was obtained from the calibrated scale associated with the ADP device. DXA assessments were performed on a Lunar Prodigy scanner (General Electric, Boston, MA, USA) with enCORE software (v.16.2). Positioning of participants was standardized using custom-made foam blocks in order to promote reliability of measurements [20]. DXA bone mineral content was divided by 0.9582 to yield an estimate of bone mineral (Mo) [21]. BIS was utilized to obtain total body water (TBW) estimates. BIS utilizes Cole modeling [22] and mixture theories [23] to predict body fluids rather than regression equations used by other impedance methods (e.g., BIA) [24]. The BIS device used in the present study (SFB7, ImpediMed, Carlsbad, CA, USA) employs 256 measurement frequencies ranging from 4 to 1000 kHz to model the *TBW* content of the body. Each participant remained in the supine position for ≥5 min immediately prior to assessment using the manufacturer-recommended hand-to-foot electrode arrangement. Duplicate assessments were performed, with the values averaged for analysis. Assessments were reviewed for quality assurance through visual inspection of Cole plots. The 4C equation of Wang et al. [25] was utilized for estimation of whole-body *FM*, as presented in the equation below. *FFM* was calculated as *BM*—*FM*, and *BF*% was calculated as (*FM*/*BM*) × 100.

FM (kg)=2.748×BV−0.699×TBW+1.129×Mo−2.051×BM

Data from our laboratory have indicated a between-day relative technical error of measurement (relative TEM; i.e., CV), of 0.6%, 1.1%, 1.4%, and 2.5% for *BM*, *FFM*, *FM*, and *BF*%, respectively, along intraclass correlation coefficients (ICC) of 0.999, 0.998, 0.986, and 0.987, respectively.

#### 2.4.3. Ultrasound Assessment

To further describe upper- and lower-body muscular adaptations, ultrasound estimates of muscle thickness of the knee extensors (MT_KE_) and elbow flexors (MT_EF_) were obtained via ultrasound with a linear-array probe connected to an android tablet (Lumify L12–4, Philips Healthcare, Amsterdam, The Netherlands). Prior to all assessments, participants remained supine for >5 min. Subsequently, transmission gel was applied to the marked measurement locations, and minimal pressure was applied by the transducer in order to avoid tissue compression. Standard depth and gain were set per manufacturer recommendations and kept consistent for all measurements at a given site within each participant. MT_KE_ was measured at the site corresponding to 50% of the length between the greater trochanter and lateral epicondyle of the femur [26], and MT_EF_ was measured at 66% of the distance from the medial acromion of the scapula to the cubital fossa [27]. A single trained technician performed all assessments, and three images were taken at each location. All images were transferred to a personal computer for analysis via ImageJ (National Institutes of Health, Bethesda, MD, USA, version 1.45 s). The distance between the superficial aponeurosis to the superior portion of the bone assessed via the straight-line function was used to determine muscle thickness and the average of the two closest MT values at each site was utilized for analysis. Data for the technician performing all ultrasound assessments indicated a between-day relative TEM (i.e., CV) of 0.7% and ICC of 0.989 for MT of the biceps brachii and a relative TEM of 3.1% and an ICC of 0.951 for MT of the rectus femoris.

#### 2.4.4. Indirect Calorimetry

Resting metabolic rate (RMR) and substrate utilization (i.e., respiratory exchange ratio [RER]) were assessed via indirect calorimetry (TrueOne 2400, ParvoMedics, Sandy, UT, USA). Gas and flow calibrations were performed each morning according to manufacturer specifications. Pre-assessment and procedural standardization were based on the recommendations of Fullmer et al. [28]. Participants were instructed to remain motionless but awake during the assessment, which took place in a climate-controlled room with the lights dimmed. The first five minutes of each test were discarded, and the assessment continued until there was a period with a coefficient of variation (CV) for RMR of ≤5%. After discarding the initial five minutes, test length was (mean ± SD) 6.16 ± 3.40 min. Data from our laboratory indicated a between-day relative TEM (i.e., CV) of 3.8% and an ICC of 0.933 for RMR estimates.

### 2.5. Nutrition Intake and Physical Activity Monitoring

#### 2.5.1. Nutritional Intake

Participants tracked their dietary intake via a multiple-pass, validated, automated self-administered 24-h dietary assessment tool (ASA24) (National Institute of Health, Bethesda, MD, USA, 2018). Under the supervision of researchers, participants completed a 24-h diet recall at baseline and post-intervention assessment visits. Throughout the study, participants were instructed to log their 24-h food intake on their own for two weekdays and one weekend day each week, throughout the 6-week intervention. A weekday was defined as Monday through Thursday and a weekend day was defined as Friday through Sunday. Participant data were downloaded from ASA24 and processed in Microsoft Excel (Microsoft, Redmond, WA, USA, 2020).

#### 2.5.2. Accelerometry

In order to objectively assess free-living physical activity levels, each participant was provided with a wrist-worn accelerometer (ActiGraph GT9X Link; Actigraph Inc., Pensacola, FL, USA) prior to the commencement of the study intervention. Participants were instructed to wear the accelerometer at all times unless bathing. The sampling rate was set at 100 Hz, and the raw data was analyzed in R using the GGIR package [29]. The physical activity variables extracted from the raw activity counts were time spent in sedentary activities, light-intensity physical activity, and moderate- or vigorous- intensity physical activity. All variables were corrected for wear time. Baseline physical activity levels were produced from pre-intervention data, while physical activity levels during the intervention were derived from valid days within the intervention period.

### 2.6. Statistical Analysis

Models were developed to predict changes in eight outcomes of interest: overall body composition (calculated as the change in 4C FFM divided by the change in BM), RMR, MT_EF_, MT_KE_, 1RM_BP_, 1RM_LP_, RTF_BP_, and RTF_LP_. To account for the large number of predictors (Table 2) and collinearity, we conducted a hierarchical cluster analysis (Figure S1), a redundancy analysis, and constructed a correlation matrix of all the variables (Figure S2) [30]. We used the results of these analyses and background knowledge to help guide variable selection by estimating the number of nonzero parameters.

We conducted the primary analyses using a two-stage approach [31]. In the first stage, using our estimate of nonzero parameters [32], we fit a series of full/reference (with all predictors) Bayesian median quantile regression models using the regularized hierarchical shrinkage “horseshoe” prior, which is a continuous global shrinkage prior that serves as a penalty function by shrinking the absolute magnitude of regression coefficients towards zero [33]. Penalization is often used in exploratory and high-dimensional settings to aid variable selection and help choose a sparser subset of predictors that contribute to model predictive performance. Predictors that were not penalized to zero or within a region of practical equivalence around it were estimated in the second stage, where we fit a series of Bayesian quantile regression models using weakly informative priors. Certain predictors were included in the second stage, regardless of whether or not the estimates were penalized to zero, such as baseline measurements of the response variable, which often had high rank correlations with the response variable.

**Figure 2:**
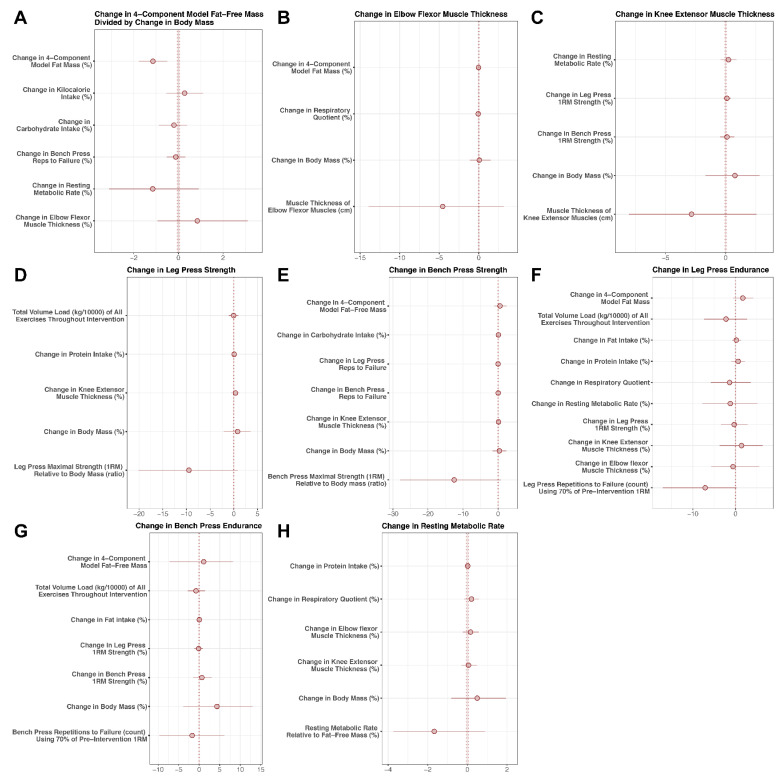
Model Coefficients. Potential predictors of changes in body composition (**A**), ultrasonography-derived muscle thickness (**B**,**C**), muscular performance (**D**–**G**), and resting metabolic rate (**H**) were examined using Bayesian regression.

**Figure 3:**
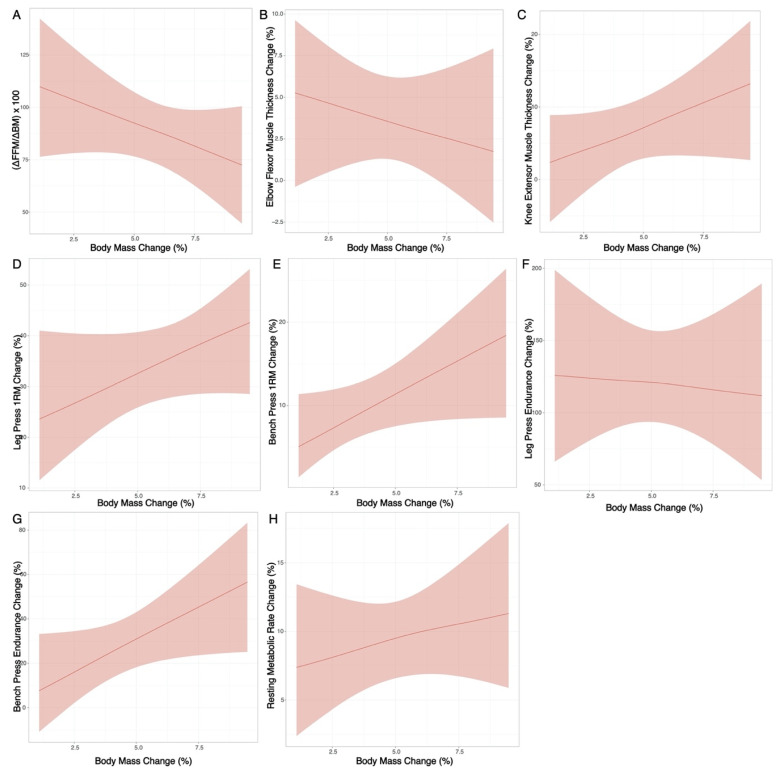
Simplified Model. A simplified regression model using only the rate of body mass gain as a potential predictor variable was generated. Outcome variables included changes in body composition (**A**), ultrasonography-derived muscle thickness (**B**,**C**), muscular performance (**D**–**G**), and resting metabolic rate (**H**).

Model coefficients (medians), standard deviations of the posterior distribution, and 95% highest density posterior intervals were reported for the predictors (Figure 2; Tables S1–S8). A simplified model using only the rate of BM change as a predictor variable was also generated (Figure 3; Table S9). Rather than dichotomize based on statistical significance or Bayes factors, we were primarily interested in estimation of relevant predictors of interest [34,35]. We plotted the 95% HDPIs and full posterior distributions with histograms (Figure S3).

We examined missing data patterns and found that the percentage of missing values across predictors and response variables varied from 0 to 53.57%, with the greatest occurrence of missing data for accelerometry variables. Some participants had missing data because they lost interest during the intervention or lacked the time to continue participation. We used multiple imputation under the missing at random (MAR) assumption to construct and analyze 60 imputed datasets with 60 iterations using the weighted predictive mean matching method [36]. Predictors and response variables were individually estimated by fitting models to each individual dataset. The posterior distributions from each of these fitted models were then combined [37]. Convergence of the imputation algorithm was inspected using trace plots to look for systematic patterns. We conducted a sensitivity analysis [38] under the missing not at random (MNAR) assumption using the delta adjustment approach [37] by supposing that individuals who left the study had smaller changes in body composition and exercise strength (Tables S10–S13). A case-complete analysis under the missing completely at random (MCAR) assumption was conducted for comparison (Tables S14–S21).

Issues in the Bayesian Markov Chain Monte Carlo algorithm were diagnosed by inspecting Gelman-Rubin statistic values, which were plotted on a histogram and by inspecting the number of model divergences and the effective sample size [39].

We assessed model fit using posterior predictive checks and assessed out of sample predictive performance using 10-fold cross validation, yielding metrics of the expected log pointwise predictive density (ELPD), which measures the predictive accuracy of the n^th^ data point taken in at a time, the Bayes R^2^ values, and the information criterion (IC) [40]. Data were analyzed in R (v. 3.6.3). The primary packages used included: stan [41], brms [42], mice [43], bayesplot [39], rms [44], boot [45], and loo [46].

## 3. Results

**Figure 4:**
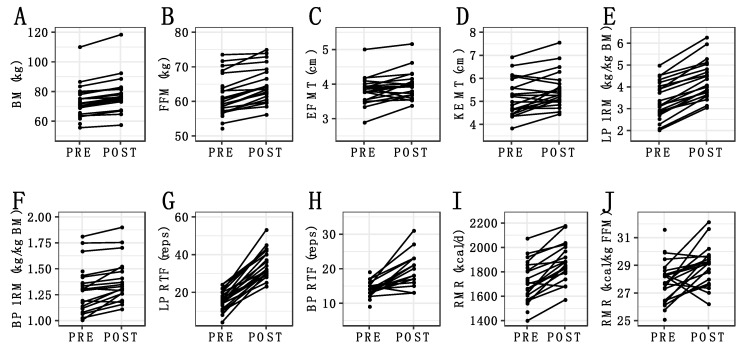
Individual Changes in Major Outcomes. The changes observed for individual participants are displayed for body mass (**A**), fat-free mass (**B**), elbow flexor muscle thickness (**C**), knee extensor muscle thickness (**D**), leg press 1-repetition maximum (**E**), bench press 1-repetition maximum (**F**), leg press repetitions to failure (**G**), bench press repetitions to failure (**H**), absolute resting metabolic rate (**I**), and resting metabolic rate relative to fat-free mass (**J**). Non-imputed data are displayed.

There were several correlations between the potential predictors (Figures S1 and S2), indicative of collinearity within the reference model with all potential predictors. Coefficients (medians) for potential predictors of each outcome are displayed in Figure 2 and presented numerically in Tables S1–S8, many of which were close to zero or too imprecise to allow for definitive conclusions. Thus, changes in the outcome variables could generally not be meaningfully predicted by the selected predictor variables. Similarly, in the simplified model using only ΔBM as a predictor variable, changes in outcomes could not be meaningfully predicted (Figure 3; Table S9). Individual changes in outcome variables are displayed in Figure 4.

Sensitivity analyses using the delta adjustment technique (Tables S10–S13) suggest that these coefficients were relatively stable to differing missing data mechanisms, even with relatively large delta adjustments. The same pattern was also generally seen with the complete case analysis. Bayes R^2^ values for the models ranged from 0.18 to 0.40 (Table 3). The absolute magnitude of ELPD and IC values increased, indicative of increasingly poorer expected predictive performance, in the order of: 1RM_BP_, MT_EF_, RMR, MT_KE_, 1RM_LP_, ΔFFM/ΔBM, RTF_BP_, and RTF_LP_.

## 4. Discussion

The purpose of this preliminary study was to examine the relationship between potential predictors of RT adaptations and changes in body composition, muscular performance, and metabolism during a 6-week period of purposeful overfeeding and RT. As expected, the intervention of progressive RT, an energy surplus, and adequate protein intake resulted in increases in whole-body FFM estimated by the 4C model (Individual Δ: +4.8 ± 2.6%) and muscle thickness estimated by ultrasonography (Individual Δ: +4.5 ± 5.9% for elbow flexors; +7.4 ± 8.4% for knee extensors) in nearly all individuals (Figure 4). Similarly, substantial improvements in muscular performance were observed. The specific adaptations to RT observed in the present study could be attributable to interactions between the RT program variables [17,47], RT- and protein-induced stimulation of muscle protein synthesis [48,49], participant characteristics (e.g., training status, age, sex, level of effort) [50], and the anabolic stimulus provided by an energy surplus [1,51]. While aspects of these factors were included as potential predictor variables of the observed RT adaptations, the statistical models ultimately could not explain the heterogeneity in outcomes within the context of the present study. Specifically, the R^2^, ELPD and IC values were indicative of relatively poor ability of the overall models to explain adaptations. Additionally, most individual predictor variables were not meaningful within these models. Similarly, the simplified models using only ∆BM as a predictor of changes in RT adaptations could not clearly explain the outcomes.

While definitive conclusions regarding the rate of BM gain cannot be made from the present investigation, the general patterns observed in the simplified ∆BM models could potentially be useful for those seeking to employ a rate of BM gain that could correspond with more favorable RT adaptations. For example, on average, a total BM gain of ~3.3% over 6 weeks (~0.55%/week) corresponded with a composition score ((∆FFM/∆BM)*100) of 100, indicative of all BM gained as FFM. However, the 95% posterior interval limits for the composition score at this rate of BM gain were ~78 to 122 (Figure 1), indicating that individuals employing this rate of BM gain could either experience simultaneous FM gain or loss while gaining FFM. While the present findings may suggest a potential relationship between slower BM accretion and positive changes in body composition and could hold utility for individuals seeking to accrue FFM while minimizing FM gain, interpretations should be made cautiously due to the wide variability observed.

Limited prior data are available concerning the degree to which the rate of BM gain during a RT program influences body composition outcomes or other adaptations. Garthe and colleagues [6] observed increases in BM, lean soft tissue, and FM in response to a caloric surplus of (mean ± SE) 544 ± 31 kcal∙day^−1^ administered through nutritional counseling, with significant improvements in maximal strength in elite athletes (>90% male) undergoing progressive RT for 8–12 weeks. Based on the reported mean values, ~63% of BM gain was attributable to lean soft tissue, on average, with the remainder gained as FM. In another study by Spillane and Willoughby [5], resistance-trained males were prescribed an additional ~1250 kcal∙day^−1^ as carbohydrate (312 g) or carbohydrate/protein/fat (196 g/94 g/22 g), which was consumed during 8 weeks of supervised RT. At baseline, both groups consumed ~1.3 g/kg of protein, while the carbohydrate group consumed only ~1.0 g/kg protein at the end of the intervention as compared to ~2.4 g/kg in the carbohydrate/protein/fat group. In the group supplementing with carbohydrate only, BM increased by 1.4 kg (1.6%) on average, with the increase attributable to increased FM (Δ: +1.5 kg; +~7.9%) rather than lean soft tissue (Δ: 0.0%). In contrast, the group supplementing with carbohydrate/protein/fat increased BM by 3.8 kg (4.5%), with apparent increases in both FM (Δ: +1.4 kg; +~9.2%) and lean soft tissue (Δ: +2.3 kg; +~3.6%). Therefore, based on reported mean values, ~0 to 61% of BM was gained as lean soft tissue depending on the group. While practically meaningful, the apparently divergent responses were not statistically significant based on traditional null hypothesis significance testing. Similarly, improvements in lower body muscular performance apparently favored the carbohydrate/protein/fat group (+16.0% in relative lower body strength vs. +9.1% in the carbohydrate supplement group), with comparable improvements of 4.5 to 5.4% in upper body strength. In another investigation, Rozenek et al. [4] reported changes in body composition and muscular strength in minimally-trained males following 8-weeks of RT, with or without consumption of a high-calorie (high carbohydrate or high protein/carbohydrate) supplement. Both supplement formulations increased BM gain (4.0 to 4.1%, on average) as compared with the RT-only control group (+0.01%, on average). Similarly, increases in FFM were apparently larger in both supplement groups (4.3 to 5.1%, on average) as compared to control (+2.1%, on average). No significant changes in FM were observed in either supplement group (Δ: −3.0 to +1.7%, on average), while a decrease of ~7.2% was observed in the RT-only group. Thus, in contrast to other investigations examining trained participants [5,6], the authors concluded that a large caloric surplus (~2000 kcal∙day^−1^) combined with progressive RT resulted in nearly all BM gained as FFM in those with minimal RT experience [4]. Interestingly, Campbell et al. [2] reported beneficial body composition changes in response to increased energy intake in aspiring female physique athletes during an 8-week RT program. Participants were assigned to a high protein group (2.5 g/kg/d) or low protein group (0.9 g/kg/d). With a modest ~400 kcal surplus created solely by increased protein consumption, the high-protein group experienced increases in BM (+1.0 kg; +1.6%) and FFM (+2.1 kg; +4.4%), along with a decrease in FM (−1.1 kg; −8.5%). In contrast, the low-protein group did not experience notable changes in BM (−0.2 kg; −0.3%), FFM (+0.6 kg; +1.2%), or FM (−0.8 kg; −6.0%).

In addition to the aforementioned investigations, three studies by Antonio et al. [7,8,9] have examined increased energy intake (~400 to 950 kcal above habitual energy intake) exclusively through higher protein consumption in resistance-trained adults. These interventions have previously been described as a form of overfeeding [3]. In these studies, which generally compared the addition of large amounts of supplemental protein to the habitual diet versus maintenance of the habitual diet without supplementation, no differences between groups were observed for FFM changes. However, a possible benefit of consuming 3.4 g/kg/d of protein, as compared to 2.3 g/kg/d, for FM reduction was observed in one study [8]. Differences in muscular performance between the high-protein and normal protein groups/conditions were not found [7,9]. The authors speculated that the lack of BM and FM gain, despite an apparent increase in energy intake relative to the habitual diet, could have been due to different training stimuli or compliance with training regimens, as well as potential effects of protein on multiple components of energy expenditure [8].

The previously discussed investigations typically employed some form of self-reported dietary record. It is well known that there are substantial limitations to self-reported energy intake (EI); in fact, some researchers have concluded that self-reported EI intake methods are “so poor that they are wholly unacceptable for scientific research on EI…” [52]. While EI was quantified via automated dietary recall in the present investigation, the notable limitations of self-reported EI were the impetus for examining changes in BM as a primary measure of dietary compliance with the hypercaloric diet rather than EI from self-report and recall methods.

In the present study, participants reported average daily protein intakes at baseline (mean: 2.3 g/kg; range: 1.4 to 5.8 g/kg) and during the intervention (mean: 2.2 g/kg; range: 1.6 to 4.8 g/kg) that are consistent with the optimal dosage for robust stimulation of MPS and subsequent increases in muscle mass when combined with an appropriate exercise training program [48,53]. In fact, the minimum value for protein intake observed for a single participant during the intervention period (1.6 g/kg) still met recommendations, with some individuals consuming much higher quantities. In addition to promoting a higher energy intake, the provision of a protein-containing dietary supplement post-exercise was based on data indicating the synergistic effects of resistance exercise and protein ingestion for stimulation of muscle protein synthesis (MPS) [49,54]. In terms of the RT stimulus, the program in the present study was designed with consideration of optimal training intensity [17], training volume [55], and training frequency [47].

A unique aspect of the present study is the a priori decision to examine the rate of BM gain as a continuous predictor variable rather than attempting to prescribe two different rates of BM gain. This approach was employed due to the expected heterogeneity in BM changes in response to RT and a hypercaloric diet. It was expected that prospectively assigning individuals to differing rates of BM gain (e.g., 0.25 vs. 0.5 kg per week) would prove difficult experimentally and that many individuals assigned to one group could have ultimately exhibited a BM gain more similar to the prescription of the other group, particularly if the difference in prescribed rates of BM gain was small. Additionally, it was deemed unnecessary, and likely inadvisable, to arbitrarily dichotomize the rate of BM gain when a spectrum of responses was expected. The present investigation has additional strengths and limitations. While previous studies have used acceptable laboratory methods of body composition assessment, including DXA [5,6], ADP [7,8,9], and hydrostatic weighing [4], each of these necessitates assumptions regarding FFM characteristics that introduce errors in both the general population [13,14] and specific athletic groups such as resistance-trained individuals [15,16]. As such, the implementation of a criterion 4C model, which is not subject to the same limitations, in the present study is a noteworthy strength. Additionally, examining both whole-body FFM changes along with muscle thickness changes of individual muscle groups contributed to greater confidence in the observed hypertrophic responses. Finally, the novel approach and rigorous analytical methods contribute to the uniqueness of the present study. Notably, we used a robust method (quantile regression) to account for extreme values and regularization via a hierarchical shrinkage prior to account for sparsity and collinearity in the reference models and used the most meaningful predictors in our second stage for estimation. Further, we accounted for the large amount of missing data in select variables using multiple imputation and examined the sensitivity of our results under various missing data mechanisms [36,37,38]. Limitations include the small sample size, short six-week duration, the inclusion of only male participants, self-reported dietary intake that was incorporated into the regression models, monitoring of crude compliance with BM gain in the non-fasted state during the intervention period, heterogeneity of the sample, and the likelihood that a much larger investigation would be needed to fully address research questions regarding meaningful predictors of heterogeneity in RT adaptations. Although not included in the present study, a control group performing resistance training without overfeeding could have helped quantify the normal variability in our outcome measures. Additionally, it is expected that training status substantially modifies the adaptations being examined. While we attempted to account for this in our models by including metrics of baseline performance, future research should examine the full spectrum of training statuses to better understand RT adaptations at different training ages.

Although practical constraints are recognized, future research in this area would benefit from the inclusion of larger samples and implementation of additional strategies to minimize missing data in order to increase the amount of meaningful information obtained. Additionally, when possible, examinations of resistance training adaptations should employ multi-component models alongside more direct estimates of muscle hypertrophy, as in the present investigation. Furthermore, the effects of overfeeding in both RT-naïve and resistance-trained populations in conjunction with longer duration RT interventions could allow for a better understanding of adaptations to these interventions. Longer interventions could also allow for the implementation of slower rates of BM gain, which could potentially benefit body composition outcomes [56]. Importantly, it remains unknown the exact amount of energy required for skeletal muscle hypertrophy, whether this cost must be met through endogenous and/or exogenous means, and which physiological factors influence this energy need [50]. A continued examination of these questions can deepen our collective understanding of adaptations to RT and help inform practical strategies to promote optimal energy surpluses and rates of BM gain when FFM accretion is desired.

## 5. Conclusions

Although definitive conclusions regarding the most influential predictors of RT adaptations during purposeful overfeeding cannot be made from this study, the present investigation represents a rigorous analysis related to a practically meaningful research question. While further work is required to more clearly define the importance of potential predictors of optimal RT adaptations, the preliminary information presented herein may prove useful for researchers and practitioners. Although a notable degree of variability was observed, a ~0.55%/week rate of BM gained corresponded with all BM being gained as FFM, on average, in the context of a six-week period of overfeeding plus RT. However, the substantial variability around this estimate indicates that prescribed rate of BM gain should be individualized with subsequent monitoring to evaluate whether or not the selected rate is consistent with the desired adaptations in a given individual.

## Figures and Tables

**Table 1:** Resistance Training Program.

Weeks 1–3			Weeks 4–6		
Week 1	Week 2	Week 3	Week 4	Week 5	Week 6
2 RIR	1 RIR	0 RIR	2 RIR	1 RIR	0 RIR
Training Sessions	Exercises	(Sets) × (Reps)	Training Sessions	Exercises	(Sets) × (Reps)
Lower Body	Hip Sled	3 × 6-8	Lower Body	Hip Sled	3 × 4-6
	Romanian Deadlift	3 × 10-12		Romanian Deadlift	3 × 8-10
	DB Walking Lunges	3 × 10-12		DB Walking Lunges	3 × 8-10
	Lying Leg Curl	2 × 10-12		Lying Leg Curl	2 × 8-10
	Leg Extension	2 × 10-12		Leg Extension	2 × 8-10
	Single Leg DB Calf Raise	2 × 10-12		Single Leg DB Calf Raise	2 × 8-10
Upper Body	Bench Press	4 × 6-8	Upper Body	Bench Press	4 × 4-6
	Supinated BB Row	3 × 6-8		Supinated BB Row	3 × 4-6
	Close Grip Bench Press	3 × 10-12		Close Grip Bench Press	3 × 8-10
	Neutral Grip Pull up	2 × 10-12		Neutral Grip Pull up	2 × 8-10
	DB Side Laterals	2 × 10-12		DB Side Laterals	2 × 8-10
	EZ Bar Bicep Curl	2 × 10-12		EZ Bar Bicep Curl	2 × 8-10
Full Body	BB Conventional Deadlift	3 × 6-8	Full Body	BB Conventional Deadlift	3 × 4-6
	Hip Sled	2 × 10-12		Hip Sled	2 × 8-10
	Seated Leg Curl	2 × 10-12		Seated Leg Curl	2 × 8-10
	Feet up Bench Press	3 × 10-12		Feet up Bench Press	3 × 8-10
	Pendlay Row	2 × 10-12		Pendlay Row	2 × 8-10
	DB Kickbacks	2 × 10-12		DB Kickbacks	2 × 8-10
	DB Curl	2 × 10-12		DB Curl	2 × 8-10

RIR: repetitions in reserve; DB: dumbbell; BB: barbell.

**Table 2:** Descriptive Data ^1,2^.

Variable	Time Point	Mean	SD	Min	Max
Body Mass (kg)	Pre	73.8	12.4	55.5	109.9
	Post	79.6	13.2	57.3	118.3
	Individual Δ (%)	4.8	2.5	1.1	9.5
Fat Mass Index (kg/m^2^)	Pre	3.2	1.3	0.6	5.8
	Post	3.4	1.6	0.4	5.9
Fat-Free Mass Index (kg/m^2^)	Pre	19.7	2.0	16.6	25.5
	Post	20.5	2.1	17.8	27.1
Muscle Thickness—Elbow Flexors (cm)	Pre	3.9	0.5	2.9	5.0
	Post	4.0	0.4	3.4	5.2
	Individual Δ (%)	4.5	5.9	−8.6	16.7
Muscle Thickness—Knee Extensors (cm)	Pre	5.2	0.8	3.8	6.9
	Post	5.4	0.8	4.4	7.5
	Individual Δ (%)	7.4	8.4	−5.4	26.4
Bench Press 1RM (kg/kg BM)	Pre	1.3	0.2	1.0	1.8
	Post	1.4	0.2	1.1	1.9
	Individual Δ (%)	12.5	8.0	4.3	35.5
Leg Press 1RM (kg/kg BM)	Pre	3.3	0.8	2.0	5.0
	Post	4.2	0.8	3.0	6.3
	Individual Δ (%)	37.2	15.7	16.5	66.7
Bench Press Endurance (reps)	Pre	14.0	2.0	9.0	19.0
	Post	19.0	5.1	13.0	31.0
	Individual Δ (%)	35.8	37.5	−13.3	106.7
Leg Press Endurance (reps)	Pre	15.4	4.7	4.0	24.0
	Post	34.9	6.8	23.0	53.0
	Individual Δ (%)	161.3	146.5	55.0	650.0
Resting Metabolic Rate (kcal/kg)	Pre	27.7	1.5	25.1	31.6
	Post	29.0	1.4	26.2	32.1
	Individual Δ (%)	10.9	6.4	−2.0	21.5
Respiratory Exchange Ratio (au)	Pre	0.8	0.1	0.7	1.1
	Post	0.8	0.0	0.8	1.0
	Individual Δ (%)	−0.6	9.2	−18.5	18.8
Energy Intake (kcal/kg)	Baseline	46.8	16.8	33.3	124.0
	Intervention	52.3	17.2	24.8	119.9
	Individual Δ (%)	15.9	30.4	−27.0	93.5
Protein Intake (g/kg)	Baseline	2.3	0.9	1.4	5.8
	Intervention	2.2	0.6	1.6	4.8
	Individual Δ (%)	11.6	30.2	−37.8	73.3
Fat Intake (g/kg)	Baseline	1.7	0.9	0.9	6.2
	Intervention	1.9	0.9	0.5	4.6
	Individual Δ (%)	16.5	36.3	−50.7	91.5
Carbohydrate Intake (g/kg)	Baseline	5.6	1.5	3.6	11.5
	Intervention	6.0	2.0	3.1	13.7
	Individual Δ (%)	11.3	34.3	−30.6	157.2
Total Volume Load (kg/10,000)	Intervention	37.1	7.4	27.4	48.8
Total Upper Body Volume Load (kg/10,000)	Intervention	16.5	3.9	11.4	25.0
Total Lower Body Volume Load (kg/10,000)	Intervention	18.4	3.5	14.4	25.1
Time in Sedentary Activity (min/day)	Baseline	635.4	66.7	481.5	740.4
	Intervention	603.5	72.9	406.6	719.1
Time in Light-intensity Activity (min/day)	Baseline	222.4	47.1	107.5	300.2
	Intervention	170.8	74.2	78.0	400.6
Time in Moderate- or Vigorous-Intensity Activity (min/day)	Baseline	113.0	43.0	0.0	198.9
	Intervention	89.1	32.3	32.8	152.3
Composition score ([∆Fat-free mass/∆Body Mass]*100)	--	90.3	36.7	8.0	145.7
Fat Mass	Individual Δ (%)	12.1	28.1	−40.4	99.4
Fat-Free Mass	Individual Δ (%)	4.8	2.6	0.5	8.8

Data from imputed dataset (*n* = 28) are displayed. ^1^ Individual Δ (%) values were calculated for each participant, with these values subsequently averaged to yield the mean individual Δ values displayed for select predictor variables in the table. Based on this calculation, the individual-level Δ value does not reflect the group-level Δ value calculated from the displayed pre- and post- group means. ^2^ Pre and post refer to the times of laboratory assessments occurring at the beginning and end of the intervention, baseline refers to data collected before the start of the resistance plus overfeeding intervention, and intervention refers to data collected during the intervention.

**Table 3:** Predictive Performance *.

	ELPD		IC			
Outcome	Est.	SE	Est.	SE	R^2^	RMSE
Composition ^†^	−146.13	5.42	292.25	10.84	0.36	45.84
∆RMR	−96.28	2.68	192.57	5.36	0.24	6.75
∆MT_KE_	−107.37	3.18	214.74	6.35	0.25	10.07
∆MT_EF_	−94.63	4.25	189.25	8.51	0.18	6.89
∆1RM_LP_	−121.07	3.89	242.14	7.78	0.34	16.30
∆1RM_BP_	−94.12	3.12	188.24	6.24	0.40	6.64
∆RTF_LP_	−179.03	11.83	358.05	23.66	0.33	147.50
∆RTF_BP_	−146.36	4.52	292.72	9.04	0.19	42.83

* Metrics reflect results of out of sample predictive performance using 10-fold cross validation. ^†^ Calculated as: (∆FFM/∆BM)*100. *Abbreviations.* ELPD: expected log pointwise predictive density; IC: information criterion; Est: estimate; SE: standard error; R^2^: Bayes R^2^; RMSE: root mean square error; RMR: resting metabolic rate relative to fat-free mass; MT_KE_: muscle thickness of knee extensors; MT_EF_: muscle thickness of elbow flexors; 1RM_LP_: one-repetition maximum for leg press; 1RM_BP_: one-repetition maximum for bench press; RTF_LP_: repetitions to failure for leg press; RTF_BP_: repetitions to failure for bench press.

## Data Availability

Data are available within the article and supplementary materials. Additional data may be available upon reasonable request to the corresponding author, pending relevant approvals.

## Supplementary Materials

The following are available online at https://www.mdpi.com/article/10.3390/jfmk6020036/s1, Figure S1: hierarchical cluster analysis, Figure S2: correlation matrix, Table S1: model coefficients for body composition, Table S2: model coefficients for muscle thickness of elbow flexors, Table S3: model coefficients for muscle thickness of knee extensors, Table S4: model coefficients for leg press maximal strength, Table S5: model coefficients for bench press maximal strength, Table S6: model coefficients for leg press endurance, Table S7: model coefficients for bench press endurance, Table S8: model coefficients for resting metabolic rate, Table S9: simplified model coefficients, Figure S3: posterior distributions, Table S10: sensitivity analysis results—body mass, Table S11: sensitivity analysis results—fat mass, Table S12: sensitivity analysis results—bench press maximal strength, Table S13: sensitivity analysis results—bench press endurance, Table S14: case-complete analysis—body composition, Table S15: case-complete analysis—muscle thickness of elbow flexors, Table S16: case-complete analysis—muscle thickness of knee extensors, Table S17: case-complete analysis— leg press maximal strength, Table S18: case-complete analysis—bench press maximal strength, Table S19: case-complete analysis—leg press endurance, Table S20: case-complete analysis—bench press endurance, Table S21: case-complete analysis—resting metabolic rate.
