# Supplementary figures and images for: Predicting Adaptations to Resistance Training Plus Overfeeding Using Bayesian Regression: A Preliminary Investigation
Source: J Funct Morphol Kinesiol. 2021 Apr 21;6(2):36. doi: 10.3390/jfmk6020036 (PMC8167794; doi:10.3390/jfmk6020036)

# Hierarchical Cluster Analysis

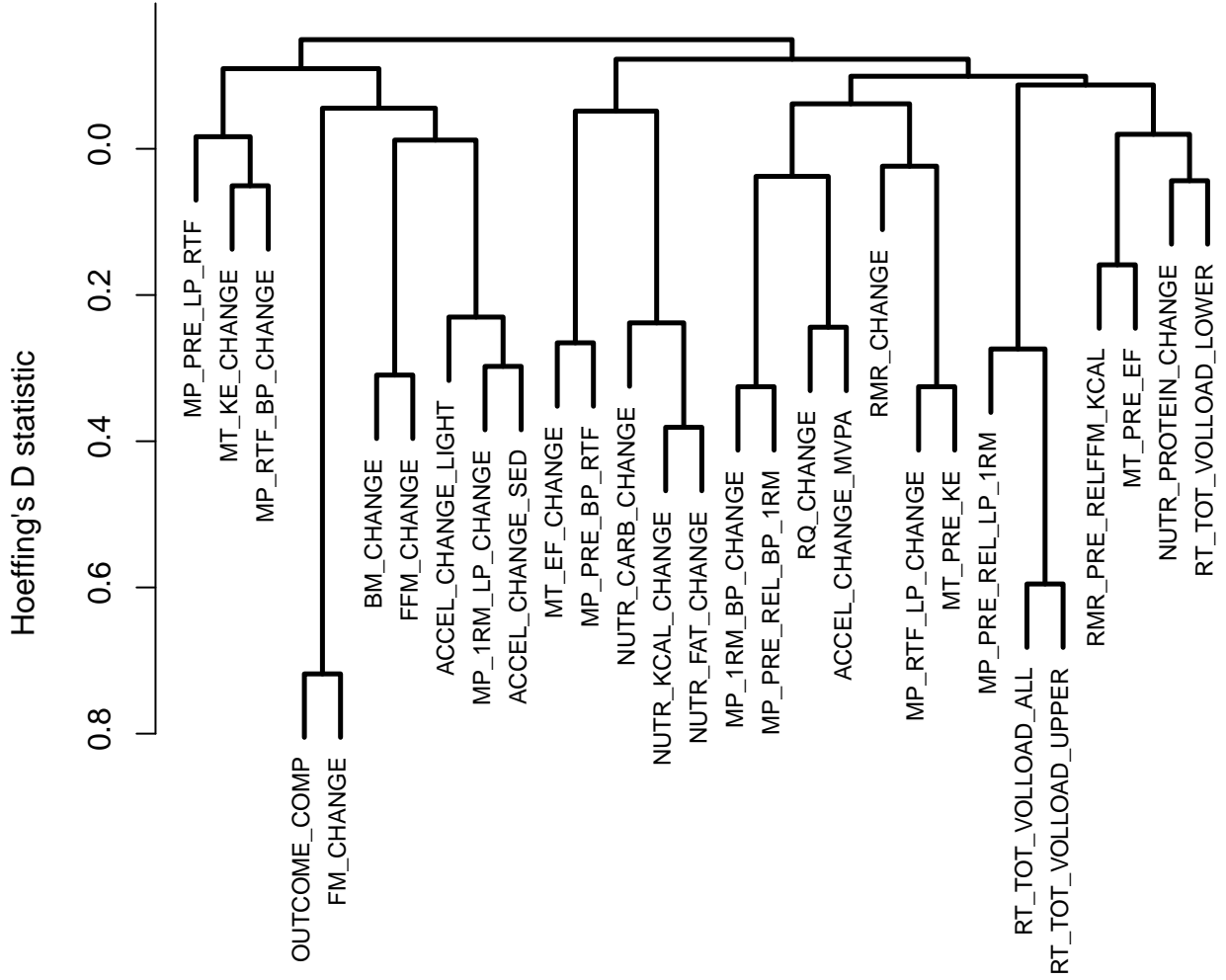

Supplement: Supplementary file 1 [file jfmk-06-00036-s001.zip › Supplemental Digital Content copy - JFMK/Figure S1.pdf]

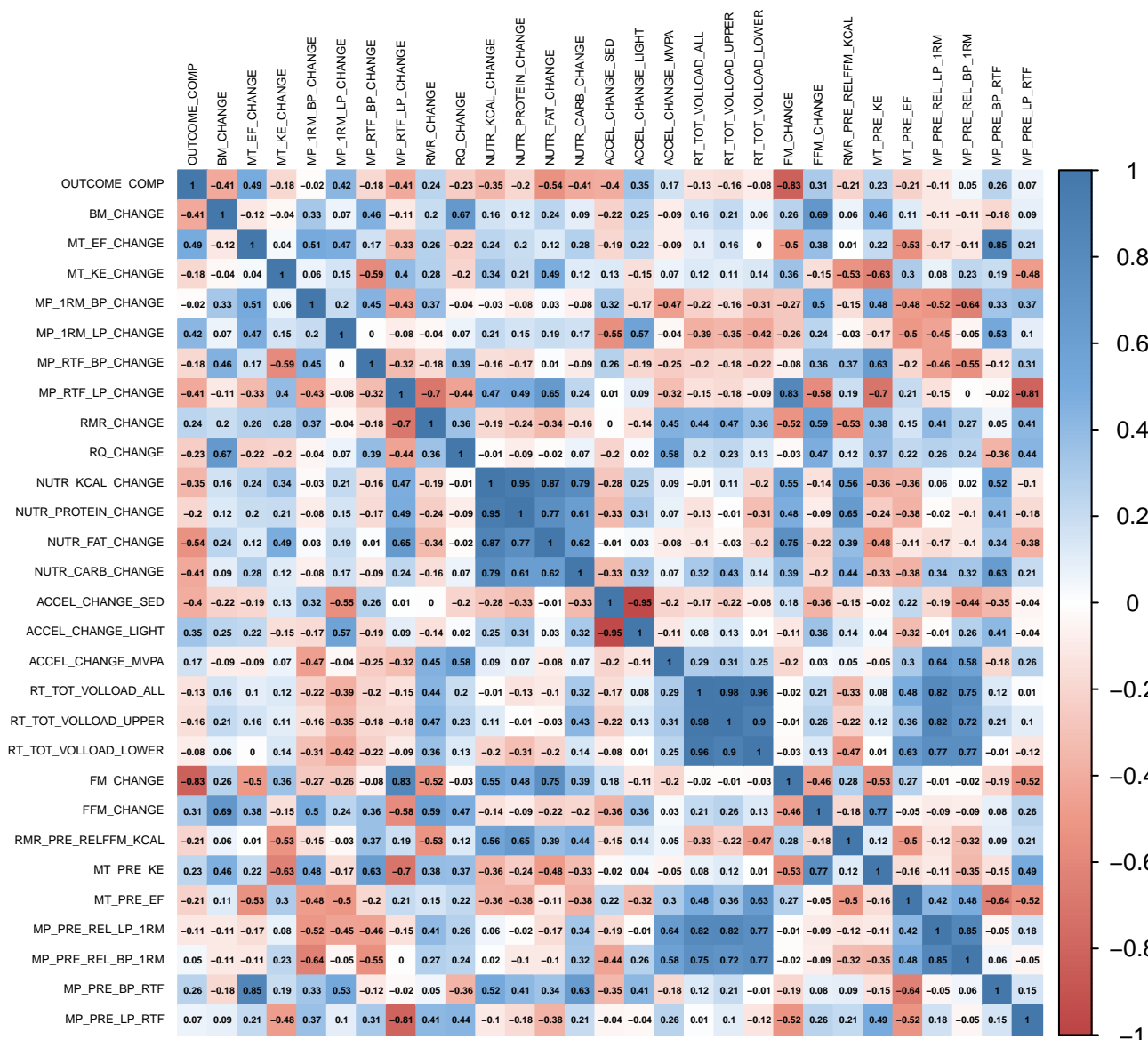

Supplement: Supplementary file 1 [file jfmk-06-00036-s001.zip › Supplemental Digital Content copy - JFMK/Figure S2.pdf]

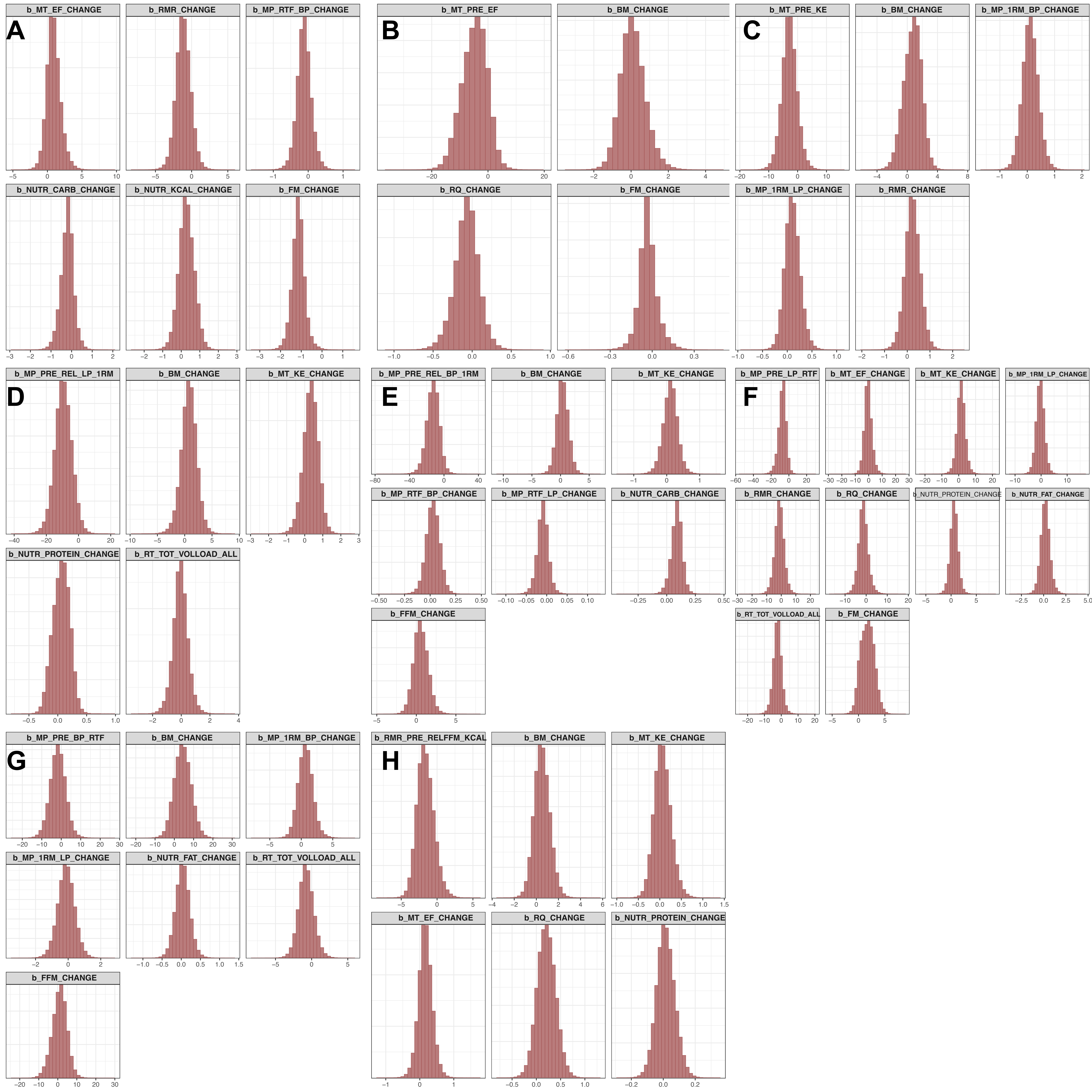

Supplement: Supplementary file 1 [file jfmk-06-00036-s001.zip › Supplemental Digital Content copy - JFMK/Figure S3.pdf]
